# Responsiveness to Vasoconstrictor Therapy in Hepatorenal Syndrome Type 1

**DOI:** 10.34067/KID.0000000000000068

**Published:** 2023-02-10

**Authors:** Juan Carlos Q. Velez, Nithin Karakala, Kasra Tayebi, Terrance J. Wickman, Muner M. B. Mohamed, Rosemary A. Kovacic, George Therapondos, Swetha R. Kanduri, Andrew S. Allegretti, Justin M. Belcher, Kevin R. Regner, Cathy Wentowski

**Affiliations:** 1 Department of Nephrology, Ochsner Health, New Orleans, Louisiana; 2 Ochsner Clinical School, The University of Queensland, Brisbane, Queensland, Australia; 3 HRS-HARMONY Consortium; 4 Division of Nephrology, Department of Medicine, University of Arkansas for Medical Sciences, Little Rock, Arkansas; 5 Multiorgan Transplant Institute, Ochsner Health, New Orleans, Louisiana; 6 Division of Nephrology, Department of Medicine, Massachusetts General Hospital, Boston, Massachusetts; 7 Division of Nephrology, Department of Medicine, Yale University, New Haven, Connecticut; 8 Veterans Affairs Connecticut Healthcare, West Haven, Connecticut; 9 Division of Nephrology, Department of Medicine, Medical College of Wisconsin, Milwaukee, Wisconsin; 10 Department of Pulmonary and Critical Care Medicine, Ochsner Health, New Orleans, Louisiana

**Keywords:** acute kidney injury and ICU nephrology, AKI, cirrhosis, ESLD, HRS-AKI, MAP, midodrine, norepinephrine, octreotide, target

## Abstract

**Key Points:**

- Raising the mean arterial pressure (MAP) during management of hepatorenal syndrome type 1 (HRS-1) is associated with improvement in kidney function, independently of baseline MAP or model for end-stage liver disease.
- Raising the MAP by 15 mm Hg or greater leads to greater reduction in serum creatinine in HRS-1.
- Norepinephrine use confers greater probability of improvement in kidney function in HRS-1 compared with midodrine/octreotide.

**Background:**

Raising mean arterial pressure (MAP) during treatment of hepatorenal syndrome type 1 (HRS-1) with vasoconstrictors (VCs) is associated with renal recovery. However, the optimal MAP target and factors associated with response to VCs remain unclear.

**Methods:**

Records from hospitalized patients with HRS-1 treated with VCs without shock were reviewed searching for those who achieved ≥5 mm Hg rise in MAP within 48 hours. We examined the relationship between the mean MAP achieved during the first 48–72 hours of VC therapy and the change in serum creatinine (sCr) up to day 14. Endpoints were >30% reduction in sCr without need for dialysis or death by day 14 (primary) or by day 30 (secondary).

**Results:**

Seventy-seven patients with HRS-1 treated for 2–10 days with either norepinephrine (*n*=49) or midodrine/octreotide (*n*=28) were included. The median age was 52 years (interquartile range [IQR], 46–60), 40% were female, and 48% had alcoholic cirrhosis. At VC initiation, median MAP was 70 mm Hg (IQR, 66–73), and median sCr was 3.8 mg/dl (IQR, 2.6–4.9). When analyzed by tertiles of mean MAP increment (5–9, 10–14, ≥15 mm Hg), there was greater reduction in sCr with greater rise in MAP (ANOVA for trend, *P* < 0.0001). By multivariate logistic regression analysis, mean MAP rise during the first 48–72 hours (odds ratio [OR], 1.15 [1.02 to 1.299], *P*=0.025), norepinephrine as VC (OR, 5.46 [1.36 to 21.86], *P*=0.017), and baseline sCr [OR, 0.63 [0.41 to 0.97], *P*=0.034) were associated with the primary endpoint, whereas mean MAP rise during the first 48–72 hours (OR, 1.17 [1.04 to 1.33], *P*=0.012) and baseline sCr (OR, 0.63 [0.39 to 0.98], *P*=0.043) were associated with the secondary endpoint.

**Conclusions:**

Greater magnitude of rise in MAP with VC therapy in HRS-1, lower baseline sCr, and use of norepinephrine over midodrine/octreotide are associated with kidney recovery. Targeting an increment of MAP ≥15 mm Hg may lead to favorable renal outcomes.

## Graphical abstract

**Figure:**
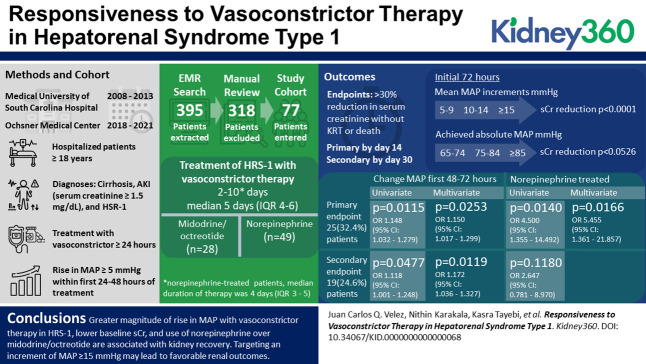

## Introduction

Vasoconstrictors (VCs) constitute the mainstay of pharmacological therapy for hepatorenal syndrome type 1 (HRS-1), a form of AKI that affects individuals with advanced cirrhosis.^1^ Lower mean arterial pressure (MAP) or reduction in MAP in patients with cirrhosis is associated with greater risk for subsequent development of HRS-1.^2-4^ Multiple lines of evidence demonstrate that the therapeutic response to VCs is directly proportional to the achieved elevation in MAP.^5-11^ The greater the magnitude in MAP increase on administration of a VC, the greater the magnitude of improvement in kidney function as determined by a reduction in serum creatinine (sCr) concentration.^8,9^ This concept applies to all VCs tested to date, including the combination of midodrine and octreotide, norepinephrine, and terlipressin.^8^ However, it remains unclear what is the optimal degree of MAP increase that maximizes efficacy. Based on trends observed in previous reports, we hypothesized that greater increases in MAP (*i.e.*, 15 versus 10 versus 5 mm Hg) may be associated with greater probability of having a favorable clinical response to VC therapy in HRS-1. In addition, we aimed to assess factors associated with improvement in kidney function in patients with HRS-1 treated with VCs who achieved a sustained rise in MAP during treatment.

## Methods

### Study Design

**Figure 1:**
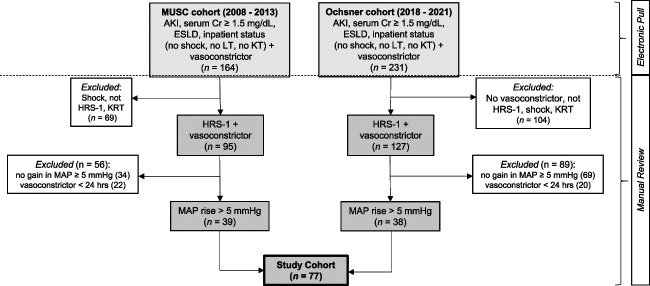
**Schematic illustrating the creation of the study cohort.** Two cohorts from two different academic medical centers were constructed with a similar objective of capturing patients with ESLD and AKI who were treated with a VC for a presumed diagnosis of HRS-1 and achieved a rise in MAP≥5 mm Hg. Because the algorithms for EMR search were not identical, different steps of manual chart review were implemented to identify patients who met the inclusion/exclusion criteria. MUSC, Medical University of South Carolina; Cr, creatinine; ESLD, end-stage liver disease; KT, kidney transplantation.

We sought to include adult patients with cirrhosis admitted to the hospital and receiving treatment with a VC for a presumed diagnosis of HRS-1. Electronic medical records (EMR) were accessed to extract all demographic and clinical data pertinent to the study. The study was composed by two cohorts. The first cohort was established at the Medical University of South Carolina Hospital between 2008 and 2013, and the second cohort was established at Ochsner Medical Center between 2018 and 2021 (Figure 1). Inclusion criteria were as follows: (*1*) hospitalized adult patients 18 years or older; (*2*) diagnosis of cirrhosis; (*3*) diagnosis of AKI by Kidney Disease Improving Global Outcomes^12^ and with a sCr≥1.5 mg/dl (threshold added to reduce the risk of including mild cases with lesser probability of representing true cases of HRS-1); (*4*) diagnosis of HRS-1; (*5*) treatment with a VC (oral midodrine and subcutaneous octreotide combination or intravenous norepinephrine) for the treatment of HRS-1 for ≥ 24 hours; and (*6*) achievement of a rise in mean daily MAP ≥5 mm Hg within the first 24–48 hours of treatment with the VC. Data of concomitant use of albumin were collected. The rationale to only include patients who achieved at least 5 mm Hg rise in MAP is that previous studies already demonstrated that a rise in MAP in the treatment of HRS-1 is more effective than no rise in MAP.^9^ In this study, the objective was to examine the degree of MAP rise necessary to achieve an optimal clinical outcome. Exclusion criteria were (*1*) use of a VC for treatment of shock; (*2*) need for RRT before the initiation of HRS-1 treatment; (*3*) status postliver transplantation (LT); (*4*) status postkidney transplantation; and (*5*) diagnosis of prior or concomitant renal parenchymal disease. All patients diagnosed with HRS-1 (HRS-AKI) met the diagnostic criteria of the International Club of Ascites.^13^ In addition, all cases of HRS-1 were further verified by having a urine sodium concentration <20 mEq/L. After exclusions generated by an automated EMR algorithm were completed, all remaining charts were manually reviewed to verify appropriateness of inclusion into the study.

### VC Therapy

Treatment of HRS-1 with VC therapy was ordered by the treating physician and implemented by the nursing personnel in the intensive care unit (ICU) (norepinephrine) or general ward (midodrine/octreotide). Drug administration was not uniformly executed as part of institutional protocols. In most instances, the goal of therapy was to achieve a rise in MAP of ≥15 mm Hg, an absolute MAP target of 85 mm Hg, or a range of absolute MAP of 80–85 or 85–90 mm Hg. Provision of the ordered treatment was affected by nurse education/understanding of the treatment rationale and variability of thresholds/directions for down-titration or up-titration of the drug dose.

### Variables

Duration of VC therapy was extracted from pharmacy records. Last day of VC therapy was considered the day when the drug was effectively discontinued or when a significant clinical event that precluded further analysis occurred (initiation of RRT, discharge to hospice care, LT, or death). Changes in MAP were assessed as absolute and relative changes over time. A minimum of four daily values (vitals as per nursing shift, patients in general wards) and a maximum of 24 daily values (hourly vitals, patients in an ICU) were collected. The baseline median MAP value corresponded to that obtained after pooling all individuals' values 24 hours before initiation of VC therapy. The baseline sCr concentration corresponded to that obtained on the morning of the day of initiation of VC therapy. Changes in sCr were assessed as absolute and relative changes over time. Only the first sCr of each day was used for the analyses.

### Endpoints

We examined the relationship between the mean change in MAP achieved (absolute and relative change, grouped by tertiles) during the first 48–72 hours of VC therapy and the corresponding change in kidney function as determined by change in sCr between baseline and the last day of follow-up after the end of VC therapy up to day 14. In addition, the relationship between MAP increase and achievement of a primary endpoint, defined as >30% reduction in sCr by the last day of follow-up after the end of VC therapy up to day 14 without need for RRT or death by day 14, was assessed. A short-term primary endpoint was chosen based on clinically meaningful and attainable endpoints used in a previously published large clinical trial in HRS-1 and taken in consideration the competing events (death, LT, hospice care) of the study population.^14^ A secondary endpoint was defined as >30% reduction in sCr without need for RRT or death by discharge by day 30. An exploratory endpoint, defined as change in slope of sCr from positive (worsening) to negative (improving) by day 7, was also examined.

### Ethics

The study was conducted with approval of the institutional review board and waiver of informed consent. The study was conducted in accordance with the Declaration of Helsinki.

### Statistics

Data were analyzed using GraphPad Prism 7 software (San Diego, CA) and R (R Foundation for Statistical Computing, Vienna, Austria) statistics software. Relationship between tertiles of MAP and change in sCr was analyzed by ANOVA for trend. Factors associated with the endpoints were analyzed by logistic regression.

## Results

From a total of 395 patients initially extracted by EMR-automated search combining both cohorts (Figure 1), 318 were subsequently excluded by manual review because of shock, diagnosis not consistent with HRS-1, prior need for RRT, no VC used, no sustained gain in MAP ≥5 mm Hg, or duration of VC therapy <24 hours. Thus, a total of 77 patients entered the study cohort (Figure 1). Patients treated for 2–10 days with either the combination of midodrine and octreotide (*n*=28), norepinephrine (*n*=49) were included. The median age was 52 years (interquartile range [IQR], 46–60), 40.3% were female, 88% were of White race (five patients were Black, two were Hispanic, and two were Asian), and 48.1% had alcoholic liver disease as etiology of cirrhosis (Table 1). At the start of VC therapy, the median baseline MAP was 70 mm Hg (IQR, 66–73), and median sCr was 3.8 mg/dl (IQR, 2.6–4.9). Median values of other pertinent laboratory tests included serum albumin 2.6 g/dl (IQR 2.0–3.3), serum sodium 131 mEq/L (IQR, 127–133), serum bilirubin 6.7 mg/dl (IQR, 3.3–20.9), model for end-stage liver disease (MELD) score 32 (IQR, 27–39), international normalized ratio 1.9 (IQR, 1.5–2.5), and platelet count 84×10^9^/L (IQR, 63–150). Median duration of VC therapy was 5 days (IQR, 4–6). Specifically for norepinephrine-treated patients, median duration of therapy was 4 days (IQR, 3–5). Seventy-five of the 77 patients (97.4%) were treated for ≥3 days, and only 2 (2.6%) were treated for 2 days. The median daily dose of midodrine was 32.5 mg (IQR, 22.5–37.5), and the median daily dose of octreotide was 300 mcg (IQR, 300–300), whereas the median dose of norepinephrine was 0.1270 mcg/kg per minute (IQR, 0.0788–0.2075) (*e.g.*, for a 75-kg patient: 9.5 mcg/min [IQR 5.9–15.6]). Forty-five of the 49 patients (92%) treated with norepinephrine had previously received midodrine/octreotide (median daily doses 37.5 mg [IQR, 30–45]/300 mcg [IQR, 300–300], respectively) and had been deemed unresponsive to the drug combination. Sixteen patients (21%) died during the index hospitalization, 23 (30%) required RRT, 16 (21%) underwent LT, and 21 (27%) were discharged to hospice.

**Table 1:** Baseline characteristics of the study cohort

Parameter	
No. of patients	77
Age (yr)	52 (46–60)
Sex (male/female)	46/31
Race (White/Black/Hispanic/Asian)	68/5/2/2
**Etiology of cirrhosis, n (%)**	
Alcoholic	37 (48)
NASH	14 (18)
HCV	11 (14)
Cryptogenic	9 (12)
Others	6 (8)
**Laboratory values**	
sCr (mg/dl)	3.8 (2.6–4.9)
Serum sodium (mEq/L)	131 (127–133)
Serum albumin (g/dl)	2.6 (2.0–3.3)
Total serum bilirubin (mg/dl)	6.7 (3.3–20.9)
INR	1.9 (1.5–2.5)
Platelet count (×109/L)	84 (63–150)
MAP (mm Hg)	70 (66–73)
MELD score	32 (27–39)
**Intravenous albumin therapy, *n* (%)**	
Before VC	67 (87)
Along with VC	65 (84)
**VC, *n* (%)**	
Midodrine-octreotide	28 (36)
Norepinephrine	49 (64)
Prior midodrine-octreotide therapy	45 (92)

Data presented as no. (%) or median (interquartile range). NASH, nonalcoholic steatohepatitis; HCV, hepatitis C virus; sCr, serum creatinine; INR, international normalized ratio; MAP, mean arterial pressure; MELD, model for end-state liver disease; VC, vasoconstrictor.

**Figure 2:**
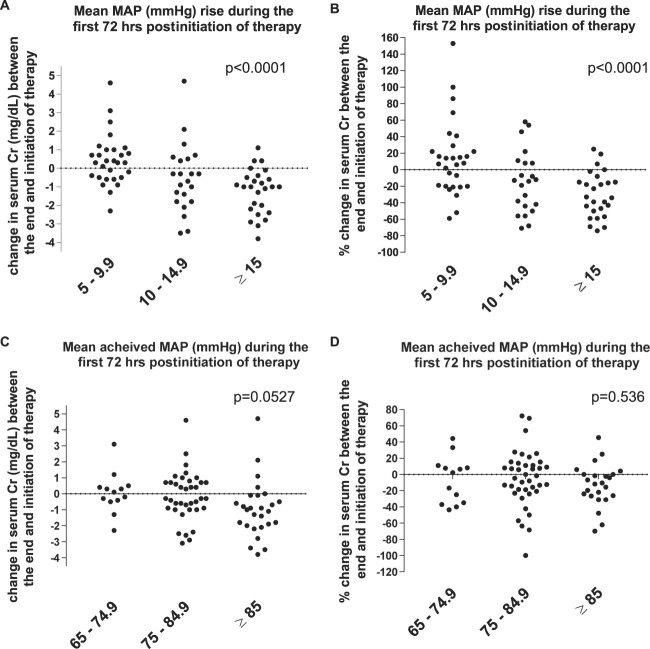
**Relationship between magnitude of MAP rise and improvement in sCr.** Tertiles of MAP rise from baseline plotted against absolute (A) and relative (B) change in sCr concentration. Tertiles on achieved MAP value plotted against absolute (C) and relative (D) change in sCr concentration. Shown *P* values for ANOVA for trend. Cr, creatinine.

When analyzed based on tertiles of mean MAP increment (5–9, 10–14, ≥15 mm Hg), there was a statistically significant trend for greater reduction in sCr with greater rise in MAP (ANOVA, *P*<0.0001). This association was observed with the assessment of variation in sCr by both absolute and relative changes (Figure 2). When analyzed based on tertiles of achieved absolute MAP (65–74, 75–84, ≥85 mm Hg), there was a nonsignificant trend for greater absolute reduction in sCr with higher absolute MAP (*P*=0.0526) (Figure 2).

The primary endpoint was met by 25 patients (32.4%), 21 norepinephrine-treated (21/49, 43%), and four midodrine/octreotide-treated (4/28, 14%). To determine factors associated with successful therapeutic response to VC therapy as assessed by the primary endpoint, we performed univariate logistic regression analysis. The influence of age, sex, MELD score, baseline sCr, baseline MAP, change in MAP during the first 48–72 hours, and used VC (norepinephrine versus midodrine/octreotide) were assessed. Change in MAP during the first 48–72 hours was significantly associated with the primary endpoint (odds ratio [OR], 1.148 [95% confidence interval (CI), 1.032 to 1.279], *P*=0.0115). Similarly, norepinephrine as VC was associated with greater probability of achieving the primary endpoint (OR, 4.50 [95% CI, 1.355 to 14.492], *P*= 0.014) (Table 2). By multivariate logistic regression analysis, both change in MAP in first 48–72 hours (OR, 1.15 [95% CI, 1.017 to 1.299], *P*=0.0253] and norepinephrine as VC (OR, 5.455 [95% CI, 1.361 to 21.857], *P*=0.0166) remained significantly associated with achievement of the primary endpoint (Table 2). Baseline MAP was not associated with the outcome. Addition of etiology of cirrhosis (alcoholic versus nonalcoholic) and total serum bilirubin to the model did not alter the results.

**Table 2:** Predictors of therapeutic response to MAP rise–targeted vasoconstrictor therapy in patients with hepatorenal syndrome type 1 defined as >30% reduction in serum creatinine without death or need for dialysis by day 14 (primary endpoint) by logistic regression analyses (*n*=77)

Variable	Univariate			Multivariate		
	OR	95% CI	*P*	OR	95% CI	*P*
Age	0.988	0.947 to 1.031	0.5843			
Male sex	0.624	0.237 to 1.640	0.3385			
MELD score	0.984	0.918 to 1.055	0.6472			
Baseline serum Cr	0.919	0.663 to 1.273	0.6101	0.633	0.410 to 0.977	0.0389
Baseline MAP	1.016	0.944 to 1.093	0.6733			
Change MAP first 48–72 h	1.148	1.032 to 1.279	0.0115^a^	1.150	1.017 to 1.299	0.0253
Norepinephrine as VC^a^	4.500	1.355 to 14.492	0.0140^a^	5.455	1.361 to 21.857	0.0166

OR, odds ratio; CI, confidence interval; MELD, model for end-stage liver disease; Cr, creatinine; MAP, mean arterial pressure; VC, vasoconstrictor.

a Versus midodrine/octreotide combination.

Regarding the timing of observing evidence of therapeutic response, among the 25 patients who met the primary endpoint, 17 (68%) started to improve after 24 hours of VC therapy as evidenced by a reduction in sCr by 0.1 mg/dl or greater; 5 (20%) began exhibiting improvement in sCr after 48 hours, 2 (8%) improved after 72 hours, and one patient did not improve until after 5 days. No serious ischemic events were recorded with norepinephrine. Sinus tachycardia requiring transition to phenylephrine occurred in one norepinephrine-treated patient. No adverse events occurred in patients treated with midodrine/octreotide.

The more stringent secondary endpoint was met by 19 patients (24.6%). MAP rise during the first 48–72 hours was significantly associated with the outcome by univariate (OR, 1.12 [1.001 to 1.248], *P*=0.048) and multivariate logistic regression (OR, 1.17 [1.04 to 1.33], *P*=0.012) (Table 3).

**Table 3:** Predictors of therapeutic response to MAP rise–targeted vasoconstrictor therapy in patients with hepatorenal syndrome type 1 defined as >30% reduction in serum creatinine without death or need for dialysis by day 30 (secondary endpoint) by logistic regression analyses (*n*=77)

Variable	Univariate			Multivariate		
	OR	95% CI	*P*	OR	95% CI	*P*
Age	0.988	0.943 to 1.035	0.6069			
Male sex	0.679	0.239 to 1.931	0.4678			
MELD score	0.985	0.914 to 1.063	0.7031			
Baseline serum Cr	0.770	0.525 to 1.130	0.1819	0.626	0.397 to 0.986	0.0434
Baseline MAP	1.013	0.936 to 1.097	0.7493			
Change MAP first 48–72 h	1.118	1.001 to 1.248	0.0477^a^	1.172	1.036 to 1.327	0.0119
Norepinephrine as VC^a^	2.647	0.781 to 8.970	0.1180			

OR, odds ratio; CI, confidence interval; MELD, model for end-stage liver disease; Cr, creatinine; MAP, mean arterial pressure; VC, vasoconstrictor.

a Versus midodrine/octreotide combination.

Regarding the exploratory endpoint of change in slope of sCr (met by 44 patients [57.1%]), change in MAP during the first 48–72 hours, and norepinephrine as VC were also significantly associated with the outcome by both univariate and multivariate analyses (Table 4). The mean MAP rise for those who achieved this exploratory endpoint was 13.5 mm Hg compared with 9.4 mm Hg for those who did not (*P*=0.0002).

**Table 4:** Predictors of therapeutic response to MAP rise–targeted vasoconstrictor therapy in patients with hepatorenal syndrome type 1 defined as change from a rising slope to a declining slope of daily change in serum creatinine (exploratory endpoint) by logistic regression analyses (*n*=77)

Variable	Univariate			Multivariate		
	OR	95% CI	*P*	OR	95% CI	*P*
Age	0.959	0.917 to 1.002	0.0629			
Male sex	0.752	0.298 to 1.899	0.5464			
MELD score	1.025	0.959 to 1.095	0.4660			
Baseline serum Cr	1.024	0.755 to 1.387	0.8806	0.624	0.409 to 0.952	0.0286
Baseline MAP	1.033	0.962 to 1.110	0.3734			
Change MAP first 48–72 h	1.248	1.105 to 1.408	0.0003^a^	1.238	1.083 to 1.416	0.0017
Norepinephrine as VC^a^	6.923	2.455 to 19.520	0.0003^a^	8.637	2.296 to 32.483	0.0014

OR, odds ratio; CI, confidence interval; MELD, model for end-stage liver disease; Cr, creatinine; MAP, mean arterial pressure; VC, vasoconstrictor.

a Versus midodrine/octreotide combination.

In addition, although sCr concentration did not significantly associate with any of the endpoints under univariate logistic regression analysis, the multivariate analyses revealed that lower sCr was associated with greater probability of achieving the primary (OR, 0.633 [95% CI, 0.410 to 0.977], *P*=0.0389), secondary (OR, 0.626 (95% CI, 0.397 to 0.986], *P*=0.043), and exploratory endpoints (OR, 0.624 (95% CI, 0.409 to 0.952], *P*=0.0286) (Tables 2–4).

## Discussion

Our study adds to an existing body of literature reporting observations demonstrating the importance of targeting an MAP increase in the treatment of HRS-1 with VCs.^8,11^ In addition to aligning with findings from previous studies, this study expands on the existing evidence by adding two specific contributions. First, although it was generally accepted that a rise in MAP with VC therapy in HRS-1 is associated with greater probability of improvement in kidney function, the minimum magnitude of the rise in MAP needed to gain benefit from the intervention has remained unclear. In this study, the greater the rise in MAP, the greater the reduction in sCr. Specifically, achievement of MAP rise ≥15 mm Hg seemed to confer additional benefit compared with a less pronounced degree of MAP rise, that is, 5–10 mm Hg above baseline.

Because a higher target of MAP rise inevitably leads to up-titration of vasopressor dosing and potential for increased risk of adverse events, a prospective randomized study targeting specific degrees of MAP rise would be needed to conclusively advocate a more aggressive MAP rise target. Nevertheless, outside of sinus tachycardia due to norepinephrine, no serious adverse events attributable to high vasopressor dosing occurred in our study. In light of the results of the CONFIRM trial and respiratory complications associated with the use of terlipressin combined with albumin, attention needs to be paid to potential pulmonary complications associated with higher vasopressor dosing.^14^ Notably, in addition to its vasopressor effect, norepinephrine has a cardiac inotropic effect^15,16^ that may reduce the risk of pulmonary edema, although conclusive data supporting such advantage are still lacking.

In clinical practice, when used for HRS-1, norepinephrine is often titrated to achieve a specific absolute MAP value (*e.g.*, 80–85 mm Hg) not necessarily taking full consideration of the baseline MAP value. Our analyses suggest that targeting a specific absolute target of MAP may not be as effective as targeting a specific increase from baseline (Figure 2). For instance, a patient with MAP 76 mm Hg at the beginning of VC therapy may only require a 9 mm Hg rise to achieve a target of 85 mm Hg, and such increase in MAP may not be sufficient to mitigate the alteration in the renal autoregulatory curve that makes individuals with HRS-1 responsive to MAP increases.^1,17,18^ For such patient, a target of 76+15=91 mm Hg may be ideal to maximize restoration of kidney perfusion and resolution of HRS-1. An observational study reported a cohort of patients with HRS-1 who increased their MAP from 79 to 93 mm Hg with norepinephrine without adverse events and with improvement in kidney function.^19^ Once again, therapeutic decisions must balance efficacy and safety; therefore, such a high MAP target may not be uniformly appropriate for all patients.

A second important contribution of our study relates to the association of MAP rise in first 48–72 hours with a categorical endpoint of improvement in kidney function. These dichotomized (yes/no) analyses commonly lead to a loss in statistical power. Nevertheless, our analyses demonstrated that a rise in MAP in the first 48–72 hours was associated with greater probability of >30% reduction in sCr without death or need for RRT by both day 14 and day 30. Considering the small size of our sample, the statistically significant ORs found in this study highlights the marked influence of MAP rise on the therapeutic response to VCs in HRS-1. Notably, 22 of the 25 patients (88%) who improved kidney function and met the primary endpoint began showing a small decrement in sCr concentration within the first 48 hours of therapy. This finding could constitute important information pertaining to the minimum duration of a trial of VC therapy in HRS-1, particularly when it relates to transferring patients to the ICU for norepinephrine infusion. While our results support the short-term benefit of MAP-targeted VC, it remains to be determined whether the observed short-term benefit could translate into improvement of harder clinical outcomes, such as need for RRT and survival beyond the 30-day mark.

Our study also showed that the use of norepinephrine as a VC was associated with greater probability of clinical benefit according to the primary and exploratory endpoints. For the treatment of HRS-1, it is generally accepted that norepinephrine has efficacy comparable with that of terlipressin^20-22^ and greater efficacy compared with midodrine/octreotide.^23^ However, head-to-head studies in HRS-1 comparing efficacy of midodrine/octreotide versus norepinephrine have not produced consistent results. One small study (*n*=23) showed comparable and unusually high efficacy (73% versus 75%),^24^ whereas another study (*n*=60) showed superiority of norepinephrine with efficacy rates more in line with other similar studies in HRS-1 (58% versus 20%, *P*=0.006).^25^ Therefore, the robust association of better clinical response to norepinephrine found in our study provides further evidence in favor of norepinephrine over midodrine/octreotide. Furthermore, the great majority of patients have failed to response to midodrine/octreotide before initiation of norepinephrine. The superiority of norepinephrine over midodrine/octreotide is primarily driven by its superior efficacy as vasopressor and potentially due to its inotropic effect.^15^ Notably, patients who did not achieve at least a 5 mm Hg rise in MAP with midodrine/octreotide were not included in the study. Therefore, our results likely underestimate the superiority of norepinephrine over midodrine/octreotide in the treatment of HRS-1.

Multivariate logistic regression analyses revealed that lower sCr was associated with greater probability of clinical benefit. The observation that higher sCr, that is, worse kidney function, associates with lesser likelihood of therapeutic response to VC is not a novel finding and aligns with the results of a recent large randomized clinical trial testing terlipressin versus placebo for the treatment of HRS-1.^14^ Not surprisingly, implementation of VC therapy in HRS-1 is more likely to result in successful clinical response when introduced early at lower levels of sCr.

A recent study explored the feasibility of administering norepinephrine for HRS-1 outside the ICU through peripheral line.^26^ Twenty patients received intravenous norepinephrine at a dose of 5–10 mcg/min targeting an increase in MAP≥10 mm Hg. The results revealed 30% of patients achieving reversal of HRS-1, a rate of response that is similar to that reported in this study. However, the study did not compare 10 versus 15 mm Hg as minimum MAP rise threshold. Another study tested a high MAP target (≥85 mm Hg) versus a low MAP (65–70 mm Hg) in a prospective, unblinded, randomized fashion using norepinephrine and vasopressin as vasopressors.^27^ The investigators did not find benefit of the high MAP target. However, because the study only included five patients with HRS-1 per treatment arm, it was deemed underpowered. In addition, they targeted absolute values of MAP and not MAP rise from baseline. Furthermore, patients in the low MAP group spontaneously exhibited MAP higher than the goal. Altogether, the totality of evidence including our study supports a MAP rise–targeted approach with VCs in HRS-1.

Our study is not without limitations. The observational and uncontrolled nature of the study limits our ability to conclusively advocate a specific MAP rise target in HRS-1 given the fact that the balance of efficacy and safety could not be properly assessed. Patients treated in the ICU may have been treated more proactively to improve their MAP. This study was not powered to assess mortality or AKI-RRT as harder outcomes. However, the short-term endpoints assessed herein are deemed clinically meaningful. Data were collected only from two academic centers. Reproducibility of the findings using a multicenter study would add validity to our observations. Notably, 37 patients (48%) in the cohort either died or were discharged to hospice. Therefore, our study population is representative of the severely ill patients with HRS-1 encountered in clinical practice. Because the great majority of norepinephrine-treated patients had previously received midodrine/octreotide, a delayed pressor effect of midodrine/octreotide is possible. However, most patients had received midodrine/octreotide for several days without evidence of improvement in the AKI. Therefore, it is unlikely that a benefit of midodrine/octreotide could have been carried over. An aspect not examined by this study was the variability among patients in the magnitude of pressor response to the VC, in other words, whether any variables were associated with different magnitude of rise in MAP in response to similar VC dose. However, factors unrelated to patient characteristics, such as treatment implementation by ICU nursing, precluded our ability to optimally examine this question. Patient-related factors affecting individual hemodynamic response to VCs deserve further investigation in a subsequent properly designed study. Despite the overall limitations, this study provides granular information about the treatment of HRS-1 with VCs that could guide practitioners in the day-to-day management of a very challenging and critically ill patient population.

In conclusion, greater magnitude of increment in MAP with VC therapy in HRS-1, lower baseline sCr, and use of norepinephrine over midodrine/octreotide are associated greater probability of short-term improvement in kidney function. Targeting an increment of MAP≥15 mm Hg may lead to favorable renal outcomes, but prospective controlled studies are still needed to define the ideal goal of VC therapy in HRS-1.

## Data Availability

All data are included in the manuscript and/or supporting information.

## References

[1] Velez JCQ Therapondos G Juncos LA. Reappraising the spectrum of AKI and hepatorenal syndrome in patients with cirrhosis. Nat Rev Nephrol. 2020;16(3):137–155. doi:10.1038/s41581-019-0218-4 31723234

[2] Zheng X Lian Y Wang P Mean arterial pressure drop is an independent risk factor of hepatorenal syndrome in patients with HBV-ACLF. Eur J Gastroenterol Hepatol. 2022;34(5):576–584. doi:10.1097/meg.0000000000002314 35131999 PMC9076250

[3] Ruiz-del-Arbol L Monescillo A Arocena C Circulatory function and hepatorenal syndrome in cirrhosis. Hepatology. 2005;42(2):439–447. doi:10.1002/hep.20766 15977202

[4] Gines A Escorsell A Gines P Incidence, predictive factors, and prognosis of the hepatorenal syndrome in cirrhosis with ascites. Gastroenterology. 1993;105(1):229-236. doi:10.1016/0016-5085(93)90031-7 8514039

[5] Nazar A Pereira GH Guevara M Predictors of response to therapy with terlipressin and albumin in patients with cirrhosis and type 1 hepatorenal syndrome. Hepatology. 2010;51(1):219–226. doi:10.1002/hep.23283 19877168

[6] Maddukuri G Cai CX Munigala S Mohammadi F Zhang Z. Targeting an early and substantial increase in mean arterial pressure is critical in the management of type 1 hepatorenal syndrome: a combined retrospective and pilot study. Dig Dis Sci. 2014;59(2):471–481. doi:10.1007/s10620-013-2899-z 24146317

[7] Cai CX Maddukuri G Jaipaul N Zhang Z. A treat-to-target concept to guide the medical management of hepatorenal syndrome. Dig Dis Sci. 2015;60(5):1474–1481. doi:10.1007/s10620-014-3483-x 25532500

[8] Velez JCQ Nietert PJ. Therapeutic response to vasoconstrictors in hepatorenal syndrome parallels increase in mean arterial pressure: a pooled analysis of clinical trials. Am J Kidney Dis. 2011;58(6):928–938. doi:10.1053/j.ajkd.2011.07.017 21962618 PMC3251915

[9] Velez JCQ Kadian M Taburyanskaya M Hepatorenal acute kidney injury and the importance of raising mean arterial pressure. Nephron. 2015;131(3):191–201. doi:10.1159/000441151 26485256 PMC4655825

[10] Angeli P Volpin R Gerunda G Reversal of type 1 hepatorenal syndrome with the administration of midodrine and octreotide. Hepatology. 1999;29(6):1690–1697. doi:10.1002/hep.510290629 10347109

[11] Boyer TD Sanyal AJ Garcia-Tsao G Predictors of response to terlipressin plus albumin in hepatorenal syndrome (HRS) type 1: relationship of serum creatinine to hemodynamics. J Hepatol. 2011;55(2):315–321. doi:10.1016/j.jhep.2010.11.020 21167235 PMC3728672

[12] Kellum JA Lameire N; KDIGO AKI Guideline Work Group. Diagnosis, evaluation, and management of acute kidney injury: a KDIGO summary (Part 1). Crit Care. 2013;17(1):204. doi:10.1186/cc11454 23394211 PMC4057151

[13] Wong F Angeli P. New diagnostic criteria and management of acute kidney injury. J Hepatol. 2017;66(4):860–861. doi:10.1016/j.jhep.2016.10.024 27984175

[14] Wong F Pappas SC Curry MP Terlipressin plus albumin for the treatment of type 1 hepatorenal syndrome. N Engl J Med. 2021;384(9):818–828. doi:10.1056/nejmoa2008290 33657294

[15] Hamzaoui O Georger JF Monnet X Early administration of norepinephrine increases cardiac preload and cardiac output in septic patients with life-threatening hypotension. Crit Care. 2010;14(4):R142. doi:10.1186/cc9207 20670424 PMC2945123

[16] Kulka PJ Tryba M. Inotropic support of the critically ill patient. A review of the agents. Drugs 1993;45(5):654-667. doi: 10.2165/00003495-199345050-00003 7686461

[17] Stadlbauer VP Wright GA Banaji M Relationship between activation of the sympathetic nervous system and renal blood flow autoregulation in cirrhosis. Gastroenterology. 2008;134(1):111–119.e2. doi:10.1053/j.gastro.2007.10.055 18166350

[18] Persson PB Ehmke H Nafz B Kirchheim HR. Sympathetic modulation of renal autoregulation by carotid occlusion in conscious dogs. Am J Physiol. 1990;258(2):F364–F370. doi:10.1152/ajprenal.1990.258.2.f364 2309893

[19] Gupta K Rani P Rohatgi A Noradrenaline for reverting hepatorenal syndrome: a prospective, observational, single-center study. Clin Exp Gastroenterol. 2018;11:317–324. doi:10.2147/ceg.s153858 30271187 PMC6151092

[20] Nassar Junior AP Farias AQ d’ Albuquerque LAC Carrilho FJ Malbouisson LMS. Terlipressin versus norepinephrine in the treatment of hepatorenal syndrome: a systematic review and meta-analysis. PLoS One. 2014;9(9):e107466. doi:10.1371/journal.pone.0107466 25203311 PMC4159336

[21] Sharma P Kumar A Shrama BC Sarin SK. An open label, pilot, randomized controlled trial of noradrenaline versus terlipressin in the treatment of type 1 hepatorenal syndrome and predictors of response. Am J Gastroenterol. 2008;103(7):1689–1697. doi:10.1111/j.1572-0241.2008.01828.x 18557715

[22] Alessandria C Ottobrelli A Debernardi-Venon W Noradrenalin vs terlipressin in patients with hepatorenal syndrome: a prospective, randomized, unblinded, pilot study. J Hepatol. 2007;47(4):499–505. doi:10.1016/j.jhep.2007.04.010 17560680

[23] Velez JCQ. Hepatorenal syndrome type 1: from diagnosis ascertainment to goal-oriented pharmacologic therapy. Kidney360. 2022;3(2):382-395. doi:10.34067/KID.0006722021 35373127 PMC8967638

[24] Tavakkoli H Yazdanpanah K Mansourian M. Noradrenalin versus the combination of midodrine and octreotide in patients with hepatorenal syndrome: randomized clinical trial. Int J Prev Med. 2012;3(11):764–769. 23189227 PMC3506087

[25] El-Desoki Mahmoud EI Abdelaziz DH Abd-Elsalam S Mansour NO. Norepinephrine is more effective than midodrine/octreotide in patients with hepatorenal syndrome-acute kidney injury: a randomized controlled trial. Front Pharmacol. 2021;12:675948. doi:10.3389/fphar.2021.675948 34276366 PMC8283260

[26] Kwong A Kim WR Kwo PY Wang U Cheng X. Feasibility and effectiveness of norepinephrine outside the intensive care setting for treatment of hepatorenal syndrome. Liver Transpl. 2021;27(8):1095-1105. doi:10.1002/lt.26065 33837624 PMC10493176

[27] Varajic B Cavallazzi R Mann J Furmanek S Guardiola J Saad M. High versus low mean arterial pressures in hepatorenal syndrome: a randomized controlled pilot trial. J Crit Care. 2019;52:186-192. doi:10.1016/j.jcrc.2019.04.006 31096099

